# Orbital reorientation in MnV_2_O_4_ observed by V NMR

**DOI:** 10.1038/s41598-017-02015-5

**Published:** 2017-05-19

**Authors:** Euna Jo, Sejun Park, Jooseop Lee, Soonchil Lee, Jeong Hyun Shim, Takehito Suzuki, Takuro Katsufuji

**Affiliations:** 1 Department of physics, Korea Advanced Institute of Science and Technology, Daejeon, 34141 Korea; 2 Korea Research Institute of Standards and Science, Daejeon, 34113 Korea; 3 Department of Physics, Waseda University, Tokyo, 169-8555 Japan; 4 Korea Institute of Science and Technology, Seoul, 02792 Korea

## Abstract

The simultaneous occurrence of the structural and magnetic phase transitions observed in MnV_2_O_4_ is one clear example of strong interplay among the spin, orbital and lattice degrees of freedom. The structure of MnV_2_O_4_ is switched by the magnetic field and the linear magnetostriction is very high. The orbital order mediates the interaction between the spin and the lattice generating these phenomena. In this work, we present experimental evidence of an orbital order in MnV_2_O_4_ and its reorientation under a rotating magnetic field as obtained by nuclear magnetic resonance(NMR). The shift in the resonance frequency of the V NMR spectrum is symmetrical with respect to 45° as an external magnetic field of 7 T rotates from the *c*-axis to the *b*-axis, indicating that the initial easy axis flips to the orthogonal direction most parallel to the field direction. The spectrum of V^3+^ ions splits into four peaks with a maximum shift of 40 MHz. Our analysis revealed that this is the combined effect of the anisotropic hyperfine field due to an ordered orbital and the dipolar hyperfine field. Reorientation of the orbital order in response to an external magnetic field accompanies the macroscopically observed magnetostriction in MnV_2_O_4_.

## Introduction

In the materials classified as Mott insulators, correlations among the spin, charge, orbital^1^ and lattice degrees of freedom yield rich phases and related physical phenomena such as metal-insulator transition^2^, colossal magnetoresistance^3^, and multiferroicity^4^. The orbital degree of freedom in transition metal ions arises when the t_2*g*_ and e_*g*_ orbitals, split from d orbitals by crystal fields, are not fully occupied. If a nondegenerate ground state is determined by the Jahn-Teller distortion, the so-called ferro-type orbital order is set where all the ions are in the same orbital state. An orbital state in one ion influences the orbital state in the neighboring ion through chemical bonding. If there remains some degeneracy in the orbital states for electrons to occupy, an orbital order other than the ferro-type can arise due to this orbital interaction. The orbital order and lattice distortion influence each other as the Jahn-Teller distortion determines the orbital order^5^. Interplay between orbital and spin orders is generated^6^ through the spin-orbit interaction in an atom and the Kugel-Khomskii interaction^7^, which determines whether the neighboring orbital state is stable for ferromagnetic or antiferromagnetic spin orders. The simultaneous occurrence of the structural and magnetic phase transitions observed in MnV_2_O_4_ is one clear example of the strong interplay among the orbital, spin, and lattice.

**Figure 1:**
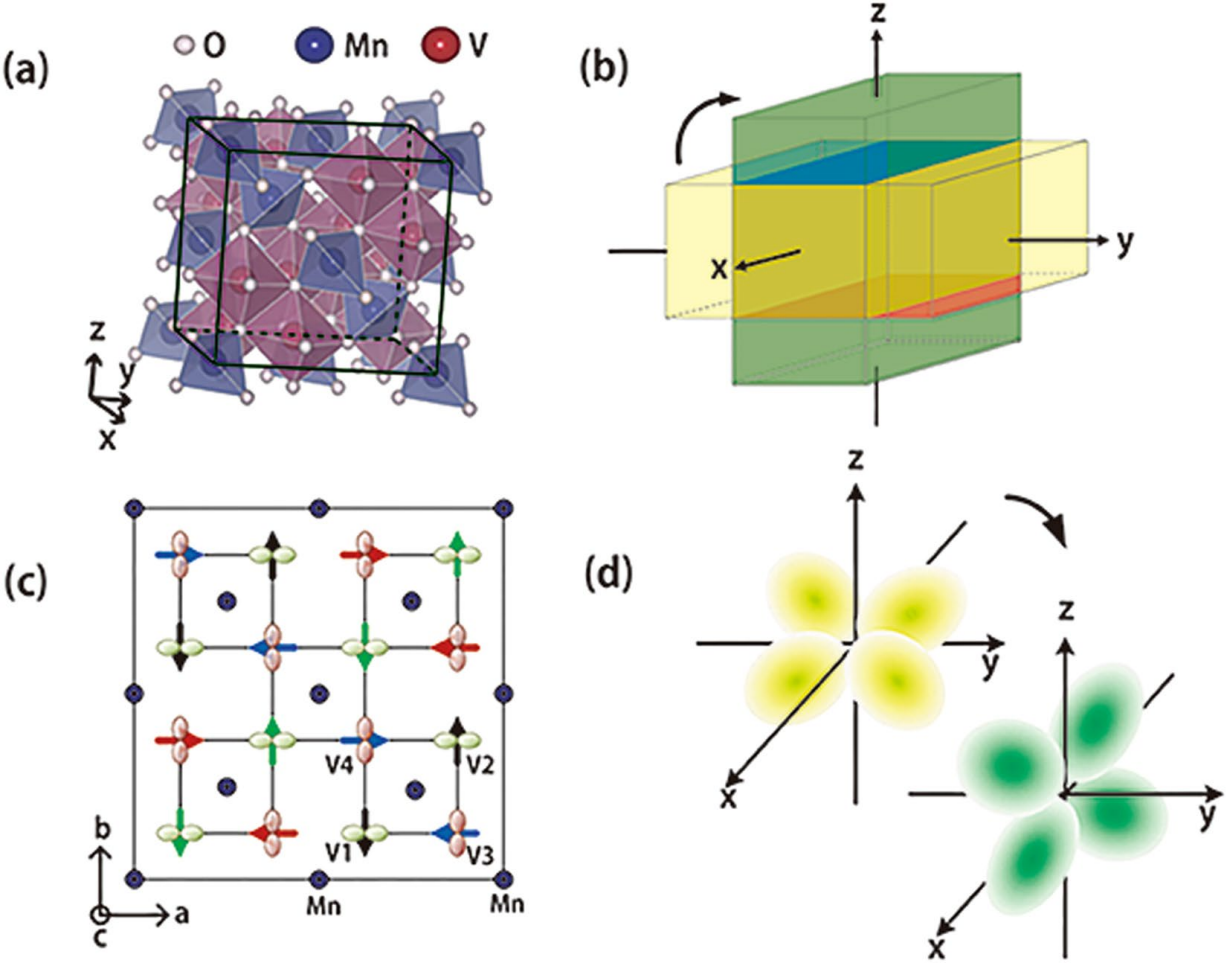
(**a**) Spinel oxide MnV_2_O_4_. The small white circles denote O^2−^ ions. Large blue circles in the tetrahedral (A) sites surrounded by four oxygen ions are Mn^2+^ ions. Large red circles in the octahedral (B) sites surrounded by six oxygen ions are V^3+^ ions. (**b**) Magnetostriction: the short axis of the tetragonal unit cell is aligned along the external field direction. The short axis flips following the net magnetic moment that rotates from the *z*-axis to the *y*-axis with the external field. (**c**) The spin and orbital states of MnV_2_O_4_ viewed along the [001] direction. The arrows represent four different directions of the V^3+^ magnetic moments, which are approximately 65° away from the axis opposite to the Mn moment aligned along the c-axis. Identically colored arrows are in the same basal plane. The blue circles represent manganese ions. (**d**) With the macroscopic easy axis flipping, the orbital state also changes the symmetry axis.

MnV_2_O_4_ has a spinel structure in which Mn^2+^ ions are in tetrahedral (A) sites surrounded by four oxygen ions at the corners, with V^3+^ ions in octahedral (B) sites surrounded by six oxygen ions(Fig. 1(a)). Mn^2+^ ions are in the 3d^5^ high spin configuration (S = 5/2) with quenched orbital angular momentum, while V^3+^ ions have the 3d^2^ spin configuration in a triply degenerate t_2*g*_ orbital. The structural change from the cubic to the tetragonal phase occurs at 53~57 K^8, 9^, almost simultaneously with the magnetic phase transition from the paramagnetic to the non-collinear ferrimagnetic state^10^. Theory also predicts an orbital order for V ions; therefore, this observation is believed to imply that the orbital order mediates the interaction between the spin and the lattice. What theory predicts is that the Jahn-Teller distortion at the VO_6_ octahedron splits the triply degenerated t_2*g*_ orbital of a V ion into a nondegenerate *xy* orbital at the ground level and doubly degenerate *yz* and *zx* orbitals at the higher energy level. One of two V electrons occupies the *xy* orbital at every V site but the second electron has freedom in the degenerate state. It can occupy *yz* and *zx* states alternatively in the antiferro-orbital order or the superposition of these states in the ferro-orbital order. The type of the orbital order in MnV_2_O_4_ has been controversial over the last decade^11–16^. The debate appears to converge by concluding that the actual state is a mixture of the real and complex orbital orders; that is, the order is somewhere between the antiferro- and ferro-orders^17, 18^. This is one reason why experiments cannot ascertain the distinct type of orbital order; another reason is that the orbital order has not been observed directly. MnV_2_O_4_ is suitable for the study of the orbital order and the lattice in the sense that no other interactions are involved in the structural distortion.

It is expected that the orbital and lattice are influenced by the external magnetic field when the spin order interacts with them. MnV_2_O_4_ provides a suitable environment for the study of the effect of the magnetic field on the orbital order because it is ferrimagnetic whereas most systems with order in the t_2*g*_ orbitals are antiferromagnetic. The structure of MnV_2_O_4_ is switched by the magnetic field near the transition and the transition temperature shifts in the magnetic field^8^, strongly supporting the claim that the simultaneous occurrence of the spin and structural phase transitions is not an accident but is instead due to the occurrence of the orbital order. The same paper also reports linear magnetostriction as large as Δ*L*/*L*~10^−3^. The easy axis of MnV_2_O_4_ is along the c-axis, which is the shortest axis of the tetragonal structure below the transition temperature. Linear magnetostriction is observed when the crystal is distorted to reduce the magnetic energy by aligning its easy axis toward the direction of the external field (Fig. 1(b)).

X-ray diffraction^11, 13^ neutron diffraction^14, 15^, and NMR^19^ measurements provided the indirect evidences of the orbital ordering in MnV_2_O_4_. In this work, we provide additional evidence of orbital ordering in MnV_2_O_4_ and show that microscopic reorientation of the orbital order occurs when the easy axis flips to generate magnetostriction effect in response to the rotation of the external magnetic field. To do this, we obtained the V NMR spectra for various directions of an external magnetic field. In the previous NMR work^19^, the spectrum was obtained at various field strengths. Their result showed that the hyperfine field is not in parallel to the spin direction, which was interpreted as a consequence of the mixed orbital state. We calculated the hyperfine field due to the orbital order as a function of the angle and compared it with the experimental data to study the state of the orbital order and its response to the external field. The symmetry of the resonance frequency shift with the field angle illustrates the easy axis flipping. The amount of the shift in the V NMR resonance frequency is only explained by the anisotropic hyperfine field due to the orbital order. The angle dependencies of the dipole field and the quadrupolar interaction were considered in the analysis of the spectral shift.

## Results and Discussions

**Figure 2:**
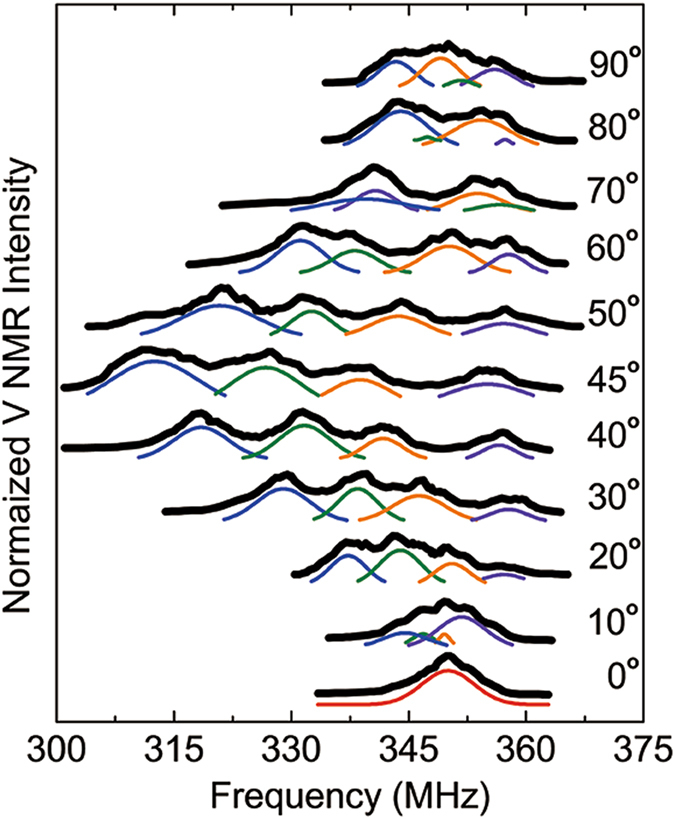
V^3+^ NMR spectra of a MnV_2_O_4_ single crystal measured at various directions of an external magnetic field of 7 T at the temperature of liquid helium. The solid curves are the gaussian fits to the decomposed peaks.

**Figure 3:**
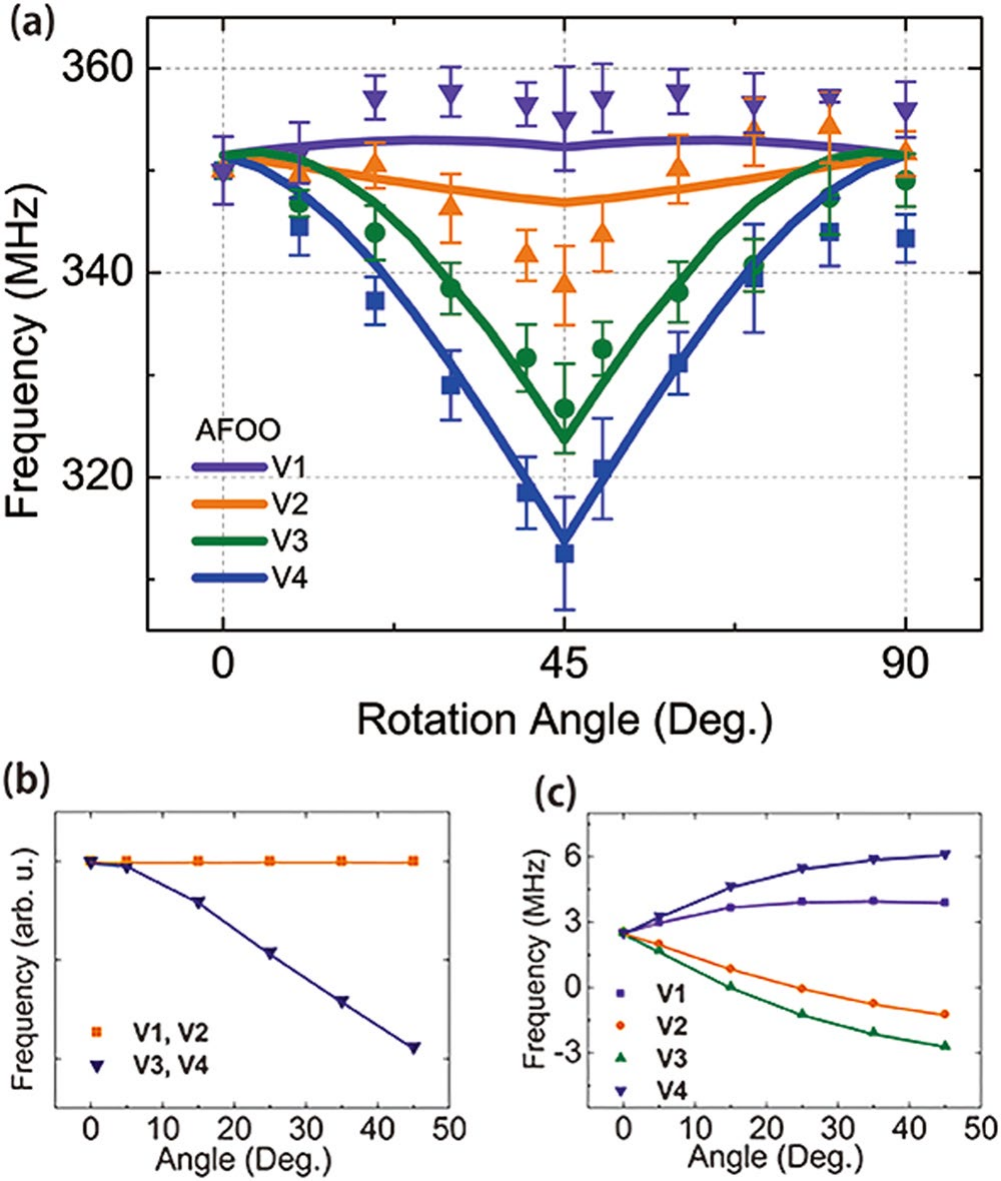
(**a**) V^3+^ NMR frequency versus the field angle. Four different symbols and colors represent the four split peaks in the V NMR spectra. The Solid lines represent the fit for the antiferro-orbital order. Frequency shifts expected for the *yz*-*zx* antiferro-orbital ordering in V ions due to (**b**) the anisotropic hyperfine field and (**c**) the dipolar field from neighboring spins. The lines in (**b**) and (**c**) are guides for the eye.

The V NMR spectra in Fig. 2 were measured as the external field of 7 T was rotated from the *c*-axis to the *b*-axis. When the magnetic field direction was along the *c*-axis of MnV_2_O_4_, the V NMR spectrum had a single peak around 350 MHz. As the angle between the magnetic field and the *c*-axis of the lattice increases, the spectrum splits into four peaks. One peak shifted toward the higher-frequency side and the other three peaks shifted toward the lower-frequency side. The maximal frequency shift occurring at 45° is 40 MHz. The center frequencies of the V NMR peaks are plotted as a function of the angle in Fig. 3. The resonance frequencies of the peaks shift with the angle until the rotation angle of the magnetic field reached 45°, at which point the peak frequencies start to go back symmetrically to their original value at 90°. This symmetry reflects the magnetostriction effect of MnV_2_O_4_. Below the transition temperature, there can be three domains of the tetragonal structures with different c-axis directions. In an external magnetic field greater than 3 T, however, only the domain structure with the c-axis parallel to the field remains^11^. Therefore, the symmetry of the NMR spectral shift implies that the initial easy axis flips to the orthogonal direction most parallel to the field direction as the external field crosses the 45° diagonal line(Fig. 1(b)).

The NMR resonance frequency of magnetic materials is determined by the vector sum of the internal and external magnetic fields, and by the quadrupolar interaction if the nuclear spin *I* > 1/2. The sources of the internal magnetic field are the on-site hyperfine field generated at a nucleus by unpaired electron spins in the same atom and the dipolar field from the spins in the neighboring atoms. The frequency shift of the V NMR spectrum cannot be explained without the anisotropic on-site hyperfine field due to the orbital order; other effects, such as the dipolar field from neighboring spins and the nuclear quadrupole interaction are too small to explain the 40 MHz shift as shown below.

When the d orbitals of magnetic ions are ordered, the hyperfine field generated at a nuclear position by the electron spin in that orbital is anisotropic. Given that the anisotropic hyperfine field is dependent on the spin direction, it can be controlled by altering the direction of the spin with an external magnetic field^20–22^. Different orbitals yield different degrees of dependence of the anisotropic hyperfine field on the spin angle. Every V^3+^ ion has one valence electron in the ground state, *xy*, and one in an excited states, *yz* or *zx*. The frequency shift of each orbital can be calculated and summed to determine the anisotropic hyperfine field for two valence electrons. There are four different spin states of vanadium ions relative to the orbital order geometry, which are labeled as V1, V2, V3, and V4 in Fig. 1(c). As the angle *θ* increases, the resonance frequency of the V ions splits. The number and behavior of the split peaks depend on the spin and orbital orders. For example, the antiferro-orbital order with Garlea’s spin model predicts that the frequency of the V1 and V2 ions remains constant while that of the V3 and V4 ions decreases as ${f}_{a}(1-3\,{\sin }^{2}\,\theta )$, where ${f}_{a}$ is a constant proportional to the anisotropic hyperfine coupling(Fig. 3(b)). The curves are in good qualitative agreement with the data representing the behavior in Fig. 3(a), except for the fact that the observed spectrum has a four-peak feature.

To analyze the additional peak splitting, the dipolar fields that a V nucleus experiences were calculated as a function of the external magnetic field angle. A V^3+^ ion is surrounded by the six nearest V spins and the six next nearest Mn spins. The dipole fields produced by the neighboring V and Mn spins at the position of a V ion were summed from the nearest to the eleventh-nearest neighbors. The distance between the central ion and these eleventh ions is shorter than the general domain size. Four V ions at different sites experience different dipolar fields as the field direction rotates from the *c*-axis to the *b*-axis. The dipolar fields of the V1 and V4 ions increased while those of the V2 and V3 decreased (Fig. 3(c)). The maximal frequency shift occurring at 45° was approximately 6 MHz. The frequency shift due to nuclear quadrupolar interaction is negligible. The typical quadrupole coupling constant ${e}^{2}qQ/h$ for a V^3+^ nuclear spin (*I* = 7/2) is approximately 3 MHz and the quadrupole frequency $\frac{\gamma }{2\pi }|{\overrightarrow{H}}_{{\rm{Q}}}|=3{e}^{2}qQ/2I(2I-1)h$ is only 0.2 MHz. In the above analysis, perfect alignment of the magnetization along the external magnetic field direction was assumed based on the previous report^19^.

These calculations indicate that the experimental data can be explained by the combined effect of the orbital order and the dipolar field, provided that the frequency shift due to the orbital order is approximately 30 MHz. We fit the experimental data to theoretical predictions with the coupling constant *f*_*a*_ as a fitting parameter. The solid lines in Fig. 3(a) show the calculation result for the antiferro-orbital order model. That is, the curves in (a) represent the sum of the calculations in (b) and (c). The fit looks somewhat satisfactory but it should be noted that the calculation is rough. First, we calculated only the component of the anisotropic hyperfine field parallel to the isotropic field as the first-order approximation assuming that the former is much smaller than the latter, which is not necessarily true. Our second assumption, based on the magnetization data^23^, was that the original spin structure remains unchanged even under a 7 T magnetic field. However, the total magnetization is not sensitive to a small amount of deformation of the spin structure, whereas this is not the case for the anisotropic hyperfine field. Moreover, we cannot exclude the possibility of incomplete alignment of the magnetization along the field direction owing to magnetic anisotropy.

The antiferro-type orbital order fit to the data a little better than the ferro-type but none of them fit perfectly. This implies that the actual state of order is the mixture of the two types. It is hard to tell how much the ferro-type order is mixed with the antiferro-type order by this simple calculation. In addition to the ferro- and antiferro-orbital models, a model based on a calculation from density functional theory was proposed^16^. In this model, the orbital states are identical at every V site but their axes alternatively rotate by 45° along and across the orbital chain, resulting in four different orbital functions with respect to one coordinate system. The four different orbital directions imply that four NMR peaks should be observed even at 0° because the c-axis, which is the symmetry axis of the spin order in a zero field, is not the symmetry axis of the orbitals in this model. This prediction clearly contradicts our observation.

Because the spectral shift of the V NMR is mostly determined by the orbital order, the symmetry in the V spectra indicates that the orbital state reorientation accompanies the easy axis flipping. If the orbitals remains unchanged, 180° symmetry would have been observed. Figure 1(d) illustrates the *xy* state, which is occupied by the electron in the ground state when the c-axis is along the z-axis direction, flips to the new ground state *zx* when the magnetic field rotates toward the y-axis as an example. The orbital states appear to be unstable near 45°, where reorientation takes place. The four peaks in the V NMR spectrum are less clear at 45° than in the spectra at 40° and 50°.

In conclusion, the V NMR spectrum splits into four peaks which are separated by a maximum of 40 MHz as the direction of the external magnetic field changes. This large shift with the angle is due to the anisotropic hyperfine field generated by the electron spins in anisotropic orbitals of the same ion. That is, the orbital of V ions is ordered. There are three domains of tetragonal structures with different c-axis directions in zero field but in an external magnetic field greater than 3 T, only the domain with the c-axis parallel to the field remains. Therefore, we expect that the easy axis flips to the orthogonal direction as the external field rotation crosses 45°. The symmetry in the V NMR frequency shift provides microscopic evidence of the actual easy axis flipping. It also indicates that the reorientation of the orbital states accompanies the easy axis flipping, or macroscopically observed large magnetostriction.

## Methods

A MnV_2_O_4_ single crystal was grown using the floating zone method. The detailed description of the sample growing process and the X-ray diffraction data to show the single crystal nature of the sample and to provide the evidence that the sample is single domain can be found in the ref. 11. ^51^V NMR spectra were obtained by measuring the spin-echo signal intensities as a function of the frequency. The capacitors in the NMR probe were carefully tuned for impedance-matching at each frequency. The crystal was initially field-cooled in the presence of a 7 T magnetic field, and then rotated around the crystallographic axes at the temperature of liquid helium. In order to control the rotation angle of the sample in the cryostat, we mounted a worm gear in the head part of the NMR probe. The gear transformed the rotation along the *z*-axis, which is parallel to the external field, outside the cryostat to the rotation along the *x*-axis inside.

The anisotropic hyperfine field owing to the orbital order is calculated as follows. The anisotropic hyperfine field is given by ref. 24

1 $${\tilde{H}}_{{\rm{ani}}}^{{\rm{on}}}=-\,g{\mu }_{B}\{\frac{-\,\overrightarrow{S}}{{r}^{3}}+\frac{\overrightarrow{r}(\overrightarrow{r}\cdot \overrightarrow{S})}{{r}^{5}}\},$$

whose expectation value is the experimentally measured field,

2 $${\overrightarrow{H}}_{{\rm{ani}}}^{{\rm{on}}}=\langle {\rm{\Psi }}|{\tilde{H}}_{{\rm{ani}}}^{{\rm{on}}}|{\rm{\Psi }}\rangle \mathrm{.}$$

If the electronic state $|{\rm{\Psi }}\rangle$ is written as $|{\rm{\Psi }}\rangle =|r\rangle |\phi \rangle |s\rangle$, where $|{r}\rangle$, $|\phi \rangle$, $|{s}\rangle$ are the radial, angular(orbital), and spin parts, respectively, then the expectation value of the anisotropic hyperfine field is given as,

3 $${\overrightarrow{H}}_{{\rm{ani}}}^{{\rm{on}}}=-\,g{\mu }_{B}\sum _{i,j}({\hat{r}}_{i}){\langle {S}_{{r}_{j}}\rangle }_{s}{\langle \frac{3{r}_{i}{r}_{j}-{r}^{2}{\delta }_{ij}}{{r}^{5}}\rangle }_{r,\phi },$$

where *r*_1_, *r*_2_, and *r*_3_ represent *x*, *y*, and *z*, respectively. A relation of angular momentum useful in calculation of the eq. (3) provided by the Wigner-Eckart theorem is

4 $$\langle lm|3{r}_{i}{r}_{j}-{r}^{2}{\delta }_{ij}|lm^{\prime} \rangle =C\langle lm|{q}_{ij}|lm^{\prime} \rangle \mathrm{.}$$

The constant *C* is independent of *m* and equals to $-\frac{2}{21}{r}^{2}$ when *l* = 2. The quadrupole moment tensor, *q*_*ij*_, is defined as

5 $${q}_{ij}=\frac{3}{2}({L}_{{r}_{i}}{L}_{{r}_{j}}+{L}_{{r}_{j}}{L}_{{r}_{i}})-{{L}}^{2}\mathrm{.}$$

For many transition metal ions, the strength of the isotropic hyperfine field is much greater than that of the anisotropic hyperfine field, because it stems from the Fermi contact interaction. Hence, as an approximation, the contribution of the anisotropic hyperfine field to NMR frequency is expressed as

6 $${\rm{\Delta }}f=\frac{\gamma }{2\pi }\langle {\rm{\Psi }}|({\overrightarrow{H}}_{{\rm{ani}}}^{{\rm{on}}}\cdot \hat{n})|{\rm{\Psi }}\rangle ,$$

in which *γ* is the nuclear gyromagnetic ratio. $\hat{n}$ is a unit vector in the direction of the isotropic hyperfine field, which is necessarily parallel to the spin direction, with the polar angle *θ* and azimuthal angle *ϕ* with respect to the c-axis.

For general applications to arbitrarily superposed 3*d* states, we calculated the matrix elements of Δ*f* on the basis of the five orbital states. The results are summarized in Table 1. All the elements are divided by the common factor *f*_*a*_ to elucidate only the angular variations. Since the anisotropic hyperfine field is dependent on the direction of a spin as expected, an orbital state can be distinguished from the others by the spin angle dependence of the hyperfine field theoretically.

**Table 1:** Angular part of the matrix ${\rm{\Delta }}f/{f}_{a}=\langle {\rm{\Psi }}|{\overrightarrow{H}}_{{\rm{ani}}}^{{\rm{on}}}\cdot \hat{n}|{\rm{\Psi }}\rangle$ for five *d* orbital states. *θ* and *ϕ* are the polar and azimuthal angles of the spin direction, respectively. Because this is a Hermitian matrix, the off-diagonal components below the diagonal are omitted.

	$|{z}^{2}\rangle$	$|{x}^{2}-{y}^{2}\rangle$	$|xy\rangle$	$|yz\rangle$	$|zx\rangle$
$\langle {z}^{2}|$	$1-3\,{\cos }^{2}\theta$	$\sqrt{3}{\sin }^{2}\theta \,\cos \,2\varphi$	$\sqrt{3}{\sin }^{2}\theta \,\sin \,2\varphi$	$-\frac{\sqrt{3}}{2}\,\sin \,2\theta \,\sin \,\varphi$	$-\frac{\sqrt{3}}{2}\,\sin \,2\theta \,\cos \,\varphi$
$\langle {x}^{2}-{y}^{2}|$	$\cdot$	$3\,{\cos }^{2}\theta -1$	$0$	$\frac{3}{2}\,\sin \,2\theta \,\sin \,\varphi$	$-\frac{3}{2}\,\sin \,2\theta \,\cos \,\varphi$
$\langle xy|$	$\cdot$	$\cdot$	$3\,{\cos }^{2}\theta -1$	$-\frac{3}{2}\,\sin \,2\theta \,\cos \,\varphi$	$-\frac{3}{2}\,\sin \,2\theta \,\sin \,\varphi$
$\langle yz|$	$\cdot$	$\cdot$	$\cdot$	$3\,{\sin }^{2}\theta \,{\cos }^{2}\varphi -1$	$-\frac{3}{2}\,{\sin }^{2}\theta \,\sin \,2\varphi$
$\langle zx|$	$\cdot$	$\cdot$	$\cdot$	$\cdot$	$3\,{\sin }^{2}\theta \,{\sin }^{2}\varphi -1$

The diagonal elements have uniaxial symmetry with respect to the axis of each orbital state. For example, the diagonal element of the $|xy\rangle$ state, Δ*f*(*xy*), can be rewritten as $3{(\hat{n}\cdot \hat{z})}^{2}-1$, in which the vector $\hat{z}$ is orthogonal to the orbital plane where the $|xy\rangle$ state locates. This interpretation is applicable to all the states. Therefore, the $|{x}^{2}-{y}^{2}\rangle$ and the $|xy\rangle$ states are indistinguishable in the sense that $|{x}^{2}-{y}^{2}\rangle$ is merely a 45° rotated state of $|xy\rangle$ around the *z* axis. However, in the limited set of 3*d* states, such as $|{z}^{2}\rangle$ and $|{x}^{2}-{y}^{2}\rangle$ in *e*_*g*_ or $|xy\rangle$, $|yz\rangle$ and $|zx\rangle$ in *t*_2*g*_, the orbital states can be distinguished from each other by the angular variation of the NMR frequency.
